# Experimental Study and Analytical Modeling on Properties of Freeze–Thaw Durability of Coal Gangue Pervious Concrete

**DOI:** 10.3390/ma16227104

**Published:** 2023-11-09

**Authors:** Yujing Wang, Junwu Xia, Pengxu Li, Linli Yu, Han Yang, Yidong Chen

**Affiliations:** 1 State Key Laboratory for Geomechanics and Deep Underground Engineering, China University of Mining and Technology, Xuzhou 221116, China; 2 Jiangsu Collaborative Innovation Center of Building Energy-Saving and Construction Technology, Jiangsu Vocational Institute of Architectural Technology, Xuzhou 221116, China; 3 School of Mechanics and Civil Engineering, China University of Mining and Technology, Xuzhou 221116, China

**Keywords:** pervious concrete, coal gangue aggregates, relative dynamic elastic modulus, permeability, grey theory, Markov process

## Abstract

To assess the freeze–thaw (F-T) durability of coal gangue pervious concrete (CGPC) in different F-T cycle media (water, 3.5 wt% NaCl solution), experimental studies on 36 groups of cube specimens and 6 groups of prismatic specimens were carried out, with designed porosity, F-T cycling media, and F-T failure times as variables. The changes in apparent morphology, mass, compressive behavior, relative dynamic elastic modulus, and permeability coefficient have been analyzed in detail. To predict the compressive strength after F-T cycles, a GM (1,1) model based on the grey system theory was developed and further improved into a more accurate grey residual–Markov model. The results reported that the cement slurry and coal gangue aggregates (CGAs) on the specimen surface continued to fall off as F-T cycles increased, and, finally, the weak point was fractured. Meanwhile, the decrease in compressive behavior and relative dynamic elastic modulus was gentle in the early phase of F-T cycles, and they gradually became faster in the later stage, showing a parabolic downward trend. The permeability coefficient increased gradually. When F-T failure occurred, specimen mass dropped precipitously. The F-T failure of CGPC was more likely to occur in 3.5 wt% NaCl solution, and the F-T failure times of samples were 25 times earlier than that of water. This study lays the foundation for an engineering application and provides a basis for the large-scale utilization of CGPC.

## 1. Introduction

The wake of the rapid advance of urbanization and economic development has led to large-scale infrastructure construction across the country, and the surface of modern cities is being gradually covered by asphalt, concrete, and other water-blocking materials [1]. Rainwater can not penetrate the ground, which increases the drainage burden of the city and leads to water accumulation on the road surface. When the rainfall is too heavy in a short period of time, it even poses a considerable threat to people’s lives and the safety of their property. This issue can be addressed effectively using pervious concrete (PC), and the aggregates of PC are only bonded by cementitious materials, which have high porosity. These structural characteristics give PC high water permeability (2–8 mm/s) [2], air permeability (15–35% for porosity) [3], maintainability, and noise reduction performance. It is conducive to improving the urban ecological environment and is the direction of future urban construction [4,5].

With the gradual improvement of research on ordinary PC, some researchers have begun to search for alternative materials [6] that can replace sand and stone as aggregates for PC, such as shells [7,8,9], ceramic particles [10], and recycled aggregates [11,12,13,14,15]. Moreover, coal gangue has gradually been used to prepare PC, and its main mineral compositions are quartz, muscovite, and kaolinite. Based on the physical and chemical characteristics of coal gangue, its use as an aggregate for concrete has been proposed, which can achieve a harmless, reduced, and environmentally friendly utilization of solid waste [16,17,18,19]. Our research team has carried out in-depth research on the preparation of CGPC for many years. By limiting the value of the permeability coefficient and compressive strength, the suitable range of the water–cement ratio, total porosity, and effective porosity was obtained. The optimum mixture ratio of CGPC was gained by combining the orthogonal test and the single factor test. The result of the research shows that the order of significance of factors affecting compressive strength and the permeability coefficient is as follows: particle size > designed porosity > water–cement ratio > PC reinforcement agent dosage. In addition, Wu et al. [20,21] prepared low-carbon and pro-environment PC by utilizing coal gangue aggregates (CGAs) and sulfoaluminate cement as cementitious materials. Compared with conventional PC, the manufacturing cost and carbon dioxide emissions were decreased to 49.4% and 43%, respectively, fully demonstrating the environmental protection and practicality of CGPC.

Nonetheless, the PC performance is limited by easy plugging and low strength, and it is more vulnerable to F-T damage in cold environments than conventional concrete. In cold areas, F-T failure of concrete structures often occurs. Therefore, further studying the impact of F-T cycles on the characteristics of PC is a crucial aspect of conducting research on PC [22,23]. Wu et al. [24] reported that rapid F-T testing is a valid approach for evaluating the F-T resistance of PC. Vancura et al. [25] demonstrated that porosity has a significant impact on the F-T resistance of PC and that there is a negative correlation between the water–binder ratio and frost resistance of PC. It has been found that the mixture proportion of coarse aggregates is an important influencing factor in the F-T resistance of PC by Kevern et al. [26]. Moreover, Yang et al. [27] presented the influences of curing conditions and environment on the F-T resistance of PC and showed that PC cured in air has poorer F-T resistance than when cured in water. In addition, they found that adding silicon powder and polypropylene fibers can effectively improve the F-T resistance of PC. In a previous study on PC by Shu et al., adding latex, an air-entraining agent, and a high-performance, water-reducing agent was demonstrated to improve the F-T resistance of PC [28]. In addition, Taheri et al. [29] demonstrated that adding a suitable amount of sand to PC can improve its performance, with a recommended sand content of 8%. Liu et al. [30] added a slight amount of sand to PC to investigate the impact of sand content on the F-T resistance of PC, and the results showed that adding a suitable quantity of sand can enhance the F-T resistance of PC. The compressive strength loss ratio of PC with a sand fraction of 5% has been the lowest since 100 cycles of F-T. In addition, every winter snowfall, in order to clear the snow, the relevant departments will also spray deicing salt on the road, and the salt and F-T cycle work together to aggravate the damage to PC structures. Yang et al. [27] reported that the existence of deicing salt would accelerate the deterioration of PC. Song et al. [31] presented that the F-T damage of PC was primarily due to the deterioration of the interface between cement and aggregate and that the F-T damage of PC inside the 3 wt% NaCl salt solution was more severe than that in the aqueous solution. Additionally, when PC is dipped in a 3 wt% NaCl solution media, it results in increased water saturation within the transition interface zone between the cement slurry and the aggregate. This heightened water saturation contributes to elevated F-T expansion stress, which, in turn, leads to a greater incidence of cracking in the interface area. A study by Tsang et al. [23] evaluated the F-T durability of PC samples in various deicing solutions, as well as in water, for comparison. They found that PC specimens that underwent F-T cycles in diverse deicers with the same mass concentration of 4%, including CaCl_2_, NaCl, and MgCl_2_, deteriorated faster than in water.

Assessing the mechanical attributes of concrete structures post-F-T cycles is crucial for those structures operating in environments subject to F-T conditions. Utilizing the principles of fatigue damage theory, a study by Yu et al. [32] formulated an equation to calculate the fatigue damage experienced by concrete subjected to F-T cycles involving both water and deicing salts. This research also led to the development of a predictive model for estimating the service lifespan of concrete in various F-T environments. Qiu et al. [33] put forward an F-T damage evolution model for coal gangue concrete, which can precisely and validly show the evolution process of the F-T deterioration of the diverse replacement rates of CGAs. Based on test data, Liu et al. [34] established an F-T damage prediction model for concrete with early F-T cycles, believing that sufficient pre-curing strength and suitable recurring conditions are necessary for concrete structures to withstand the early F-T cycles. Up to now, most studies in the field of F-T damage have only focused on ordinary concrete and ordinary PC, and mature theories such as hydrostatic and osmotic pressure have been formed. Furthermore, the grey prediction model [35,36] and nonlinear curve fitting have been used to predict the change in PC performance after F-T. Despite the importance of understanding F-T cycles, there is a notable lack of published studies specifically examining their effects on CGPC. Moreover, existing predictive models for assessing CGPC behavior post-F-T cycles are somewhat limited. Given these gaps, it becomes essential to investigate how F-T cycles influence the properties of CGPC, both for environmental sustainability and to broaden its range of applications.

In the existing literature, although considerable research has been dedicated to the mechanical properties and water permeability of pervious concrete, few studies have explored the behavior of CGPC under Freeze–Thaw (F-T) cycles, especially when subjected to different F-T media. Furthermore, applications based on grey theory models like GM (1,1), grey residual models, and grey residual–Markov models for predicting the compressive strength of CGPC after F-T cycles remain largely unexplored. This present study aims to fill these gaps by conducting systematic experiments to evaluate the decay law of mechanical properties (mass loss rate, compressive behavior, and relative dynamic elastic modulus) and the change law of the water permeability of CGPC during F-T cycles under different F-T media (water and 3.5 wt% NaCl solution). Our research introduces and validates grey residual–Markov models for compressive strength. This study can enrich the theoretical system relating to the damage of CGPC under F-T cycles and provide theoretical support for the application of CGPC under F-T conditions.

## 2. Materials and Methods

### 2.1. Materials

**Figure 1:**
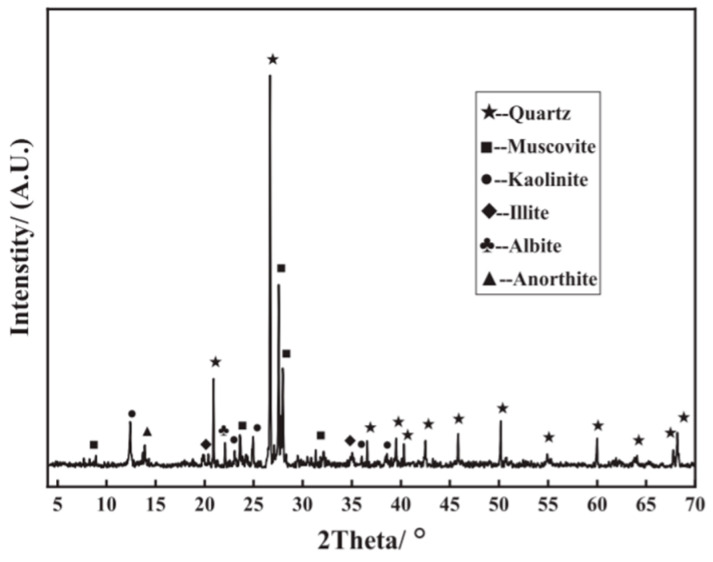
XRD spectrum of the coal gangue.

In this test, ordinary Portland cement (P.O.52.5) was applied as the cementitious materials, and its chemical analysis and properties, which were tested based on the Chinese GB175-2007 standard [37], are displayed in Table 1 and Table 2, respectively. Jiajing Ecological Engineering Technology Co., Ltd. in Huaian, China provided a PC reinforcement agent. Its content is 3.2%, and it has a fineness of 3.51% and a density of 2.44 g/cm^3^. The raw coal gangue was made in the Zhangshuang Lou mine in Xuzhou, Jiangsu Province, China, and the CGAs are sorted with a particle size of 9.5–16 mm using a vibrating screen after being crushed by a jaw crusher. Referring to the relevant requirements in GB/T14685-2022 [38], “Pebbles and Crushed Stone for Construction”, the basic physical properties of CGAs with a particle size of 9.5–16 mm are tested, as shown in Table 3. The primary chemical constituents of the sample were identified through X-ray fluorescence (XRF) analysis, the results of which are detailed in Table 4. The dominant chemical elements were found to be SiO_2_, constituting 61.7%, and Al_2_O_3_, comprising 19.11%. Additionally, X-ray diffraction (XRD) testing revealed the principal mineral components of the CGAs to be quartz, muscovite, and kaolinite, as shown in the XRD spectrum presented in Figure 1. According to the classification relationship between the chemical composition of coal gangue and the type of rock, the coal gangue used in the test is a kind of sandstone coal gangue similar to natural aggregate [17].

Sodium chloride was produced by Xilong Science Co., Ltd. in Shantou, China. In this test, the water–cement ratio was determined to be 0.29 through earlier work.

### 2.2. Sample Preparation and Mixture Proportions

In this study, 36 groups of cube specimens (100 mm × 100 mm × 100 mm) and 6 groups of prismatic specimens (100 mm × 100 mm × 400 mm) were designed. Among them, the samples tested in each research were six sets of cube specimens, and six sets of prismatic specimens were used to observe the apparent morphological changes of specimens after F-T cycling. In addition, each group uses three specimens for testing, and the final result is the arithmetic mean of these tests. If an error exceeds 15%, it is discarded. Three variables, including designed porosity (18%, 20%, and 22%), F-T cycling media (pure water, 3.5 wt% NaCl solution), and F-T cycles times (0, 25, 50, 75, 100, and 125), were considered to investigate the change in the properties of CGPC. In this experiment, P and D represent the design porosity and the times of the F-T cycles, respectively, and W and N represent water and 3.5 wt% NaCl solution, respectively. For instance, WP18D25 and NP18D25 represent specimens with a designed porosity of 18% that have undergone 25 F-T cycles in water and 3.5 wt% NaCl salt solution, respectively.

The volumetric method was used for the proportioning design of CGPC according to the target porosity, and the mix proportion of CGPC was displayed in Table 5. The calculation formula is below.

(1) $$\frac{M_{G}}{\rho_{g}}+\frac{M_{C}}{\rho_{c}}+\frac{M_{W}}{\rho_{w}}+P=1,$$

where *M_G_*, *M_C_*, and *M_W_* are CGA mass, cement mass, and water mass per unit volume (kg/m^3^), respectively, and $\rho_{g},\;\;\rho_{c}$, and $\rho_{w}$ are the density of CGA, cement, and water (kg/m^3^), and *P* is designed porosity (%).

CGA mass per unit volume was calculated via the following equation:

(2) $$M_{G}=\alpha \cdot \rho_{g},$$

where *α* is the correction factor of coal gangue coarse aggregate mass; moreover, it is a dimensionless parameter, and the value of *α* is 0.98. $\rho_{g}$ is the compacted bulk density of the coal gangue coarse aggregate.

**Figure 2:**
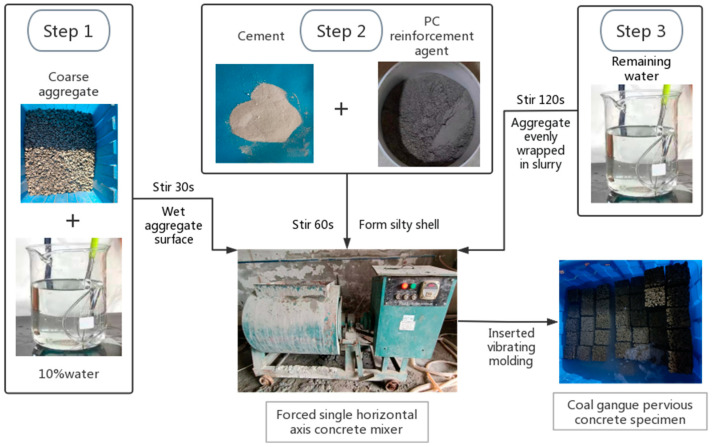
Preparation sequence of CGPC.

Ordinary concrete is mainly prepared using a one-step charging operation, and its workability is better than PC. Combined with the preliminary test, it was found that the performance of the CGPC specimen prepared by the coarse aggregates enveloped with cement paste method is better than that using the one-step charging operation, and the preparation sequence of CGPC is displaced in Figure 2. The molding method adopts the method of inserted vibrating molding. Specifically, the thoroughly mixed PC is evenly divided into three parts and loaded into the mold. Each time the mold is loaded, a vibrating rod is used to insert and vibrate 20 times from the outside to the inside. During the insertion, the force should be uniform and the insertion frequency should be the same.

### 2.3. Test Methods

#### 2.3.1. F-T Testing

In accordance with the requirements of the quick-freezing method in Chinese standard GB/T 50082-2019 [39], a fast TDR-28-type concrete F-T testing machine was utilized to conduct this F-T test. After being cured in water for 28 days, the CGPC specimen, with a size of 100 mm × 100 mm × 400 mm, is placed into the test box in the fast-freezing machine. The F-T medium is injected into the test box, and the solution height is ensured to be 2–3 cm higher than the specimens. Then, the test box is placed into the F-T case in the fast-freezing machine in order. Then, holes are drilled for the temperature-measuring specimen, which is placed in the center of the F-T case. The central temperature-measuring sensor is then placed in the hole of the temperature-measuring specimen. Other temperature-measuring sensors are evenly placed on the diagonal line of the F-T case. Then, the parameters of the quick-freezing machine are set, and the minimum and maximum temperature of the specimen center are set to −18 °C and 5 °C, respectively. It is essential to ensure that each F-T cycle time is 120–240 min, and that the melting time is not less than a quarter of the time required for an F-T cycle. The F-T test is started after activating the machine once the F-T cycle time is set to 25 times. When each 25 F-T cycle finishes, the specimen box is removed from the F-T cycle test case, and the F-T media inside the specimen box is poured out. Then, the specimen is removed, and the water and scum on the surface of the specimen are wiped for testing. Upon completion of each test cycle, the sample is returned to its specimen container to guarantee uniform freezing. The F-T medium is then replenished to proceed with the next F-T cycle. The testing is concluded when any one of the following three criteria is met: (1) the CGPC specimen experiences a mass loss rate of 5%; (2) the number of F-T cycles reaches 125; or (3) the relative dynamic elastic modulus declines to 60%.

#### 2.3.2. Mass Loss Rate Test

This mass loss rate test is performed as per the Chinese standard GB/T 50082-2019 [39]. After being cured in water for 28 days, the CGPC specimen is taken out, and its initial mass *m*_0_ is measured. The mass of the specimen *m_n_* is weighed after each of the 25 F-T cycles. Before measurement, the surface scum of the concrete sample should be cleaned, the surface moisture should be wiped dry, and, then, its external damage should be checked.

The mass loss rate is obtained as follows:

(3) $$n=\frac{m_{n}-m_{0}}{m_{0}}\times 100\%,$$

where *m*_0_ and *m_n_* represent the mass of samples before and after the F-T cycle test (g), respectively; *n* is the mass loss rate (%).

#### 2.3.3. Compressive Strength Test

The compressive behaviors of CGPC were conducted using a hydraulic pressure testing machine. Before formal loading, the specimen is pre-compressed, and, during formal loading, the loading speed is set to 0.3 MPa/s. To ensure uniform pressure, the side of the formed specimen is used as the pressure-bearing surface. Each group uses three test blocks for testing, and the final result is the arithmetic mean of these tests. If an error exceeds 15%, it is discarded. The formula for calculating compressive strength is as follows:

(4) $$f_{cu}=0.95\frac{F_{max}}{S},$$

where $F_{max}\;is\;peak\;load\;of\;specimen\left(N\right),\;f_{cu}$ is cubic compressive strengths (MPa), and S is the compressed surface area (${mm}^{2}$).

#### 2.3.4. Water Permeability Test

**Figure 3:**
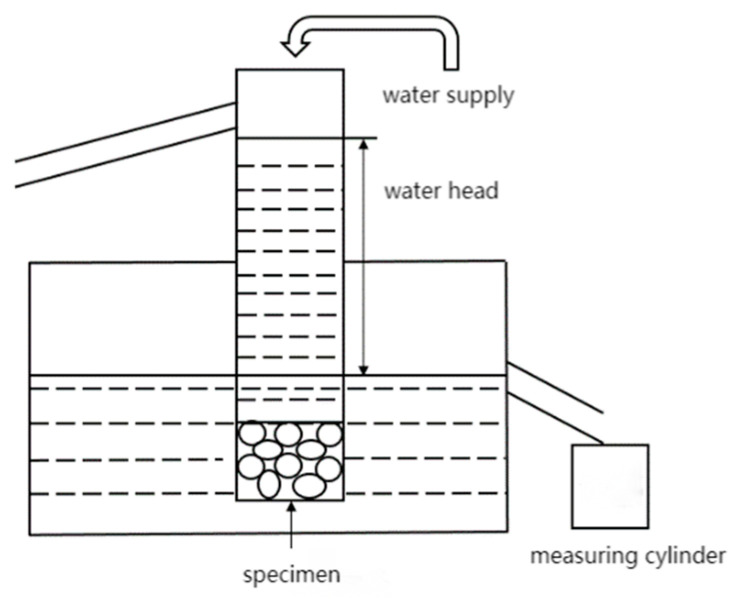
Diagrammatic sketch of water permeability test device.

In line with Chinese standard CJJ/T135-2009 [40] “Technical Specifications for PC Pavement”, the testing method of the permeability coefficient adopts the fixed water level method, and a diagrammatic sketch of the water permeability test device is shown in Figure 3.

**Figure 4:**
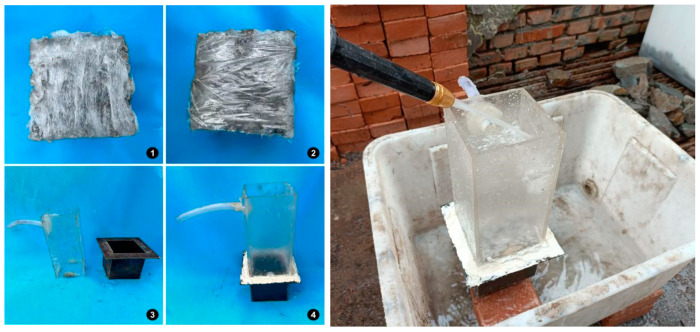
Test procedure of permeability coefficient. (**1**) specimen with a 10 cm wide PVC film; (**2**) specimen with a 10 cm wide PVC film in three layers (**3**) the acrylic board and iron box; (**4**) using AB adhesive to bond the acrylic board and iron box together2.3.5. Relative Dynamic Elastic Modulus (RDEM) Test.

The test procedures of the permeability coefficient are shown in Figure 4. First, evenly apply vaseline to the four sides of the CGPC specimen to ensure that all holes are blocked. Secondly, wrap the specimen with a 10 cm wide PVC film in three layers to ensure tight wrapping; thirdly, place the test piece wrapped in cling film from below into a self-made permeability coefficient-measuring instrument, and use AB adhesive to bond the acrylic board and iron box together to ensure no water leakage; then, use plasticine to block the pores between the surface of the PC specimen and acrylic plate, place the instrument into the water tank, and adjust the water inflow of water pipe to ensure the stability of the water level difference; then, measure the water temperature (*T*) in the tank and the water head *H* between the water level in transparent acrylic and the water level in the water tank; finally, measure the amount of water passing through the test piece in *t* seconds using a steel ruler. The arithmetic mean of three amounts of water is taken as *Q*. The permeability coefficient of the specimen can be obtained using the following formula:

(5) $$K_{T}=\frac{QL}{AHt}\cdot \frac{\eta_{T}}{\eta_{15}},$$

where $K_{T}$ is the permeability coefficient (mm/s) of specimens with a water temperature of *T* °C; *Q* is water seepage (mm^3^); *L* is test piece thickness (mm); *A* is surface area (mm^2^); $\frac{\eta_{T}}{\eta_{15}}$ is relative viscosity; *H* is water head (mm), and *t* is test duration (s).

In this research, the RDEM of CGPC is tested with an ultrasonic non-metallic detector, which is a non-metallic ultrasonic detector produced by Beijing Zhibolian Company. The ultrasonic wave velocity of the specimen is tested via an indirect test.

**Figure 5:**
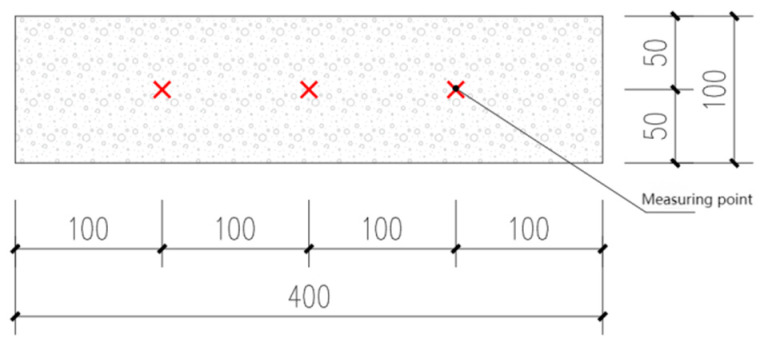
Diagrammatic sketch of measuring point position.

The test steps of the dynamic elastic modulus of CGPC are as follows: First, open the instrument setting, select the test module, and set the parameters. Then, apply vaseline on the probe of the instrument and zero, apply vaseline near the measuring point of the CGPC, and place the probe near the measuring point to measure its ultrasonic wave velocity. Three equidistant measuring points on the side of the specimen are selected for each specimen for testing, as illustrated in Figure 5. The ultrasonic wave velocity of the specimen is then measured, and its REDM is calculated. The arithmetic average of the three calculated results is obtained as the final result, and the value with an error exceeding 15% is discarded. The formula for the RDEM is as follows:

(6) $$P_{i}=\frac{E_{dni}}{E_{d0i}}\times 100\%=({\frac{V_{pni}}{V_{p0i}})}^{2}\times 100\%,$$

where $P_{i}$ is the RDEM of the F-T cycles (%), $E_{dni}$ and $V_{pni}$ are the dynamic elastic modulus (GPa) and ultrasonic longitudinal wave velocity (km/s) of the specimen after the F-T cycles, respectively, and $E_{d0i}$ and $V_{p0i}$ represent the dynamic elastic modulus (GPa) and ultrasonic longitudinal wave velocity (km/s) of the samples before the F-T process, respectively.

## 3. Experiment Results and Discussions

### 3.1. Apparent Morphology Changes of Specimen

**Figure 6:**
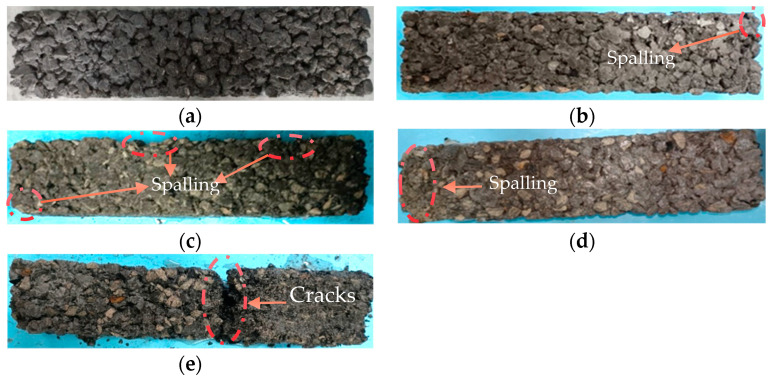
Apparent morphology changes of CGPC under F-T cycles: (**a**) NP18D0; (**b**) NP18D50; (**c**) NP18D75; (**d**) NP18D100; (**e**) NP18D125.

Figure 6 displays the apparent morphological changes of CGPC under F-T conditions with a designed porosity of 18% in 3.5 wt% NaCl solution. As can be seen from Figure 6, the F-T failure process of CGPC specimens generally conforms to the three macroscopic failure processes of PC, namely, the separation of surface coarse CGAs from cement slurry, the shedding of coarse CGAs, and the generation and development of cracks. The apparent changes in other groups are similar, and only F-T cycles are different when F-T failure occurs.

In the initial stages of the F-T process, there were no significant changes in the visual morphology of the CGPC specimens. However, after 50 F-T cycles, the corner cement of the CGPC samples began to gradually detach. As the number of F-T cycles increased, portions of the CGAs became visible on the surface of the CGPC specimens. By the time 75 F-T cycles were completed, both the cement slurry and CGAs had started to dislodge from the ends and corners of the samples. After reaching 100 F-T cycles, it can be observed that a large area of CGAs was exposed, and that part of the cavity appeared on the surface of the specimen. The phenomenon of slag and CGAs spalling at both ends of the specimen was serious. The apparent morphological changes in this study were similar to those in the study by Xiang et al. [4]. After 125 F-T cycles, the weak part of the CGPC specimens was broken, the CGAs fell off in a large range, and the CGPC specimen was damaged. The shedding of these pastes and aggregates is the main reason for the mass loss [4]. Therefore, as the number of F-T cycles increased, CGPC specimen deterioration occurred gradually. With an increase in the design porosity of the CGPC specimens, the F-T resistance of the CGPC specimens decreased by several degrees. This is because the specimens with larger design porosity have more internal pores and the cement slurry content inside the specimens, as well as the bonding force between CGAs and CGAs, is less.

During the experiment, it was found that the CGPC specimens with a designed porosity of 18% and 20% underwent 125 F-T cycles entirely in water before failure occurred, and, among them, 18% of the CGPC specimens maintained good integrity. Meanwhile, the CGPC specimens with a designed porosity of 22% underwent 75 F-T cycles in water before failure occurred. Moreover, samples with a designed porosity of 18% and 20% underwent 100 F-T cycles in a 3.5 wt% NaCl solution. In contrast, specimens with a designed porosity level of 22% only completed 50 F-T cycles in 3.5 wt% NaCl solution. CGPC specimens in 3.5 wt% NaCl salt solution experienced F-T failure earlier than in water, indicating that chloride environments significantly impact F-T failure in CGPC specimens.

### 3.2. Mass Loss

**Figure 7:**
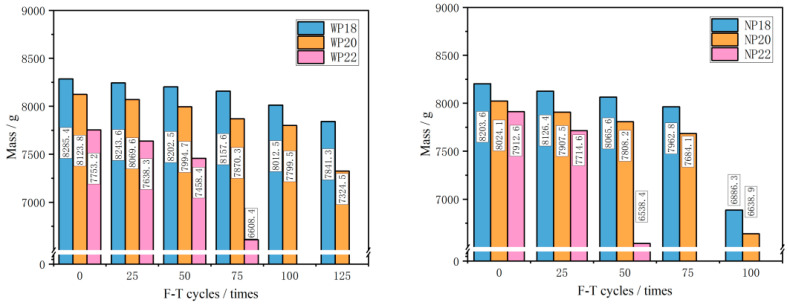
Mass of CGPC under F-T cycle.

Mass loss is an important index used to measure the deterioration degree of CGPC under F-T cycles. It can be observed from Figure 7 that the mass of CGPC decreases as the number of F-T cycles increases. When CGPC specimens were damaged, the CGPC was broken, and part of the CGA fell off. Moreover, its quality dropped dramatically with the mass loss rate exceeding 5%, which means the CGPC specimen incurred F-T damage. During the test, it was found that the samples in the groups of P18 and P20 had good F-T resistance, and they underwent 125 F-T cycles in water and 100 F-T cycles in 3.5 wt% NaCl solution, respectively. In addition, the WP18 specimen still maintained good integrity after 125 F-T cycles, but its mass loss rate exceeded 5%, which was considered to have reached the F-T failure standard. The WP20 and NP20 specimens also maintained good integrity after being taken out, but a fracture occurred during mass weighing. Moreover, the specimen of P22 was fractured after 75 F-T cycles in water, and the specimen mass was greatly reduced. As shown in Figure 7, with an increase in the design porosity of the CGPC, the deterioration degree of the specimen gradually increases during F-T cycles. The reason for this is that, with the design porosity of CGPC increasing, the cement paste content in the specimen was less, and the bonding force between adjacent CGA was also less, resulting in the poor F-T resistance of CGPC.

**Figure 8:**
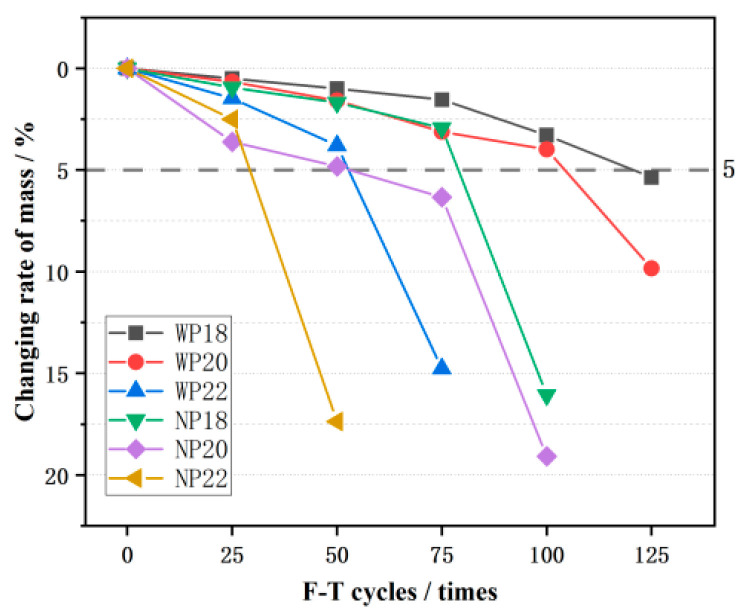
Mass loss rate of CGPC under F-T cycles.

By comparing the mass loss rate of CGPC in different F-T environments, it was found from Figure 8 that the times of the F-T damage of specimens in 3.5 wt% NaCl solution were less than that in water. The specimen in group NP22 had the fastest damage speed, and the damage occurred within 50 F-T cycles. The sample with group WP22 was damaged after 75 F-T cycles. Specimens in groups NP18 and NP20 were damaged during 75–100 F-T cycles, while those in groups WP18 and WP20 were damaged during 100–125 F-T cycles, demonstrating that the times of F-T failure in salt solution were lower than that in water. However, it is important to note that, in the absence of F-T conditions, NaCl does not have a significant negative effect on concrete because Freidel’s salt, produced by the reaction of NaCl and cement slurry, is not a very destructive component for concrete. The higher mass loss rate in the NaCl solution is not caused by the chemical attack of NaCl. Adding NaCl gives pervious concrete more hygroscopic properties and higher saturation, allowing it to reach critical saturation faster, and the expansion pressure generated when freezing is higher [4,31].

### 3.3. Compressive Behavior

**Figure 9:**
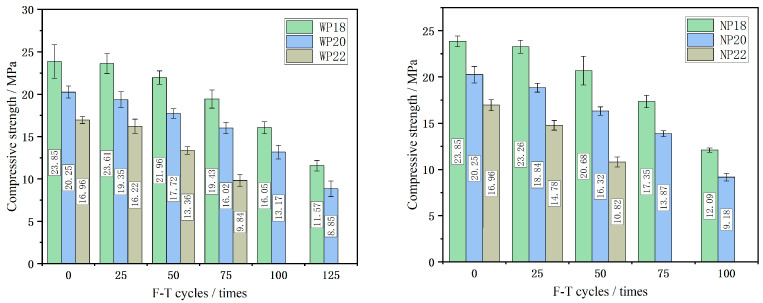
Compressive strength of CGPC under F-T cycle.

One key metric for assessing the suitability of a concrete structure for its intended service conditions is its residual compressive behavior during F-T cycles. To this end, the compressive performance of CGPC post-F-T cycles was examined. Figure 9 illustrates that, with the progression of F-T cycles, there was a gradual reduction in the compressive strength of CGPC, accompanied by an incremental increase in the rate of strength loss. Notably, during the initial 0–25 F-T cycles, the compressive strength of the CGPC remained relatively stable. When other conditions were equal, the deterioration degree of the compressive strength of CGPC increased as the design porosity increased. This is because there are increasingly more pores in the specimen, and the F-T resistance of the specimen was worse as the design porosity increased.

**Figure 10:**
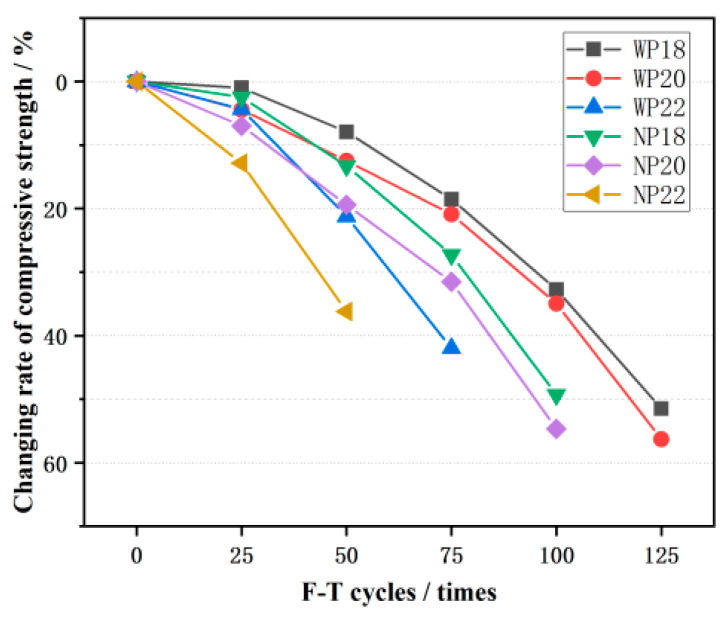
Change rate of compressive strength of CGPC under F-T cycles.

Furthermore, Figure 10 displays the change rate of the compressive strength of CGPC during the F-T process. The compressive strength loss rate curve of both the WP20 and NP20 groups fell faster compared with that in groups WP18 and NP18, but the compressive strength loss rate of the CGPC in group P22 increased more than that in group P20. Moreover, the compressive strength loss rates of NP18, NP20, and NP22 were 13.3%, 19.4%, and 38.5% after 50 F-T cycles, respectively. Additionally, the compressive strength loss rates of WP18, WP20, and WP22 were 18.5%, 20.9%, and 41.9%, respectively. The reason for this phenomenon may be that, when the design porosity of the CGPC specimens prepared in this test is 18% and 20%, the damage form of the CGPC during the compressive test is mainly CGA failure. While the design porosity is 22%, the failure mode of the CGPC specimen in the compressive test is mainly the failure of the aggregate cement bonding surface, and the F-T resistance of the CGPC is slightly poor. Furthermore, it can be concluded that the effect of F-T cycles on compressive behavior is similar to its effect on mass change.

The extent of compressive behavior degradation in CGPC also varies depending on the F-T environment. CGPC exposed to a 3.5 wt% NaCl solution exhibited greater deterioration compared to those subjected to water after the same number of F-T cycles. As illustrated in Figure 10, the rates of compressive strength loss for the WP18 and NP18 samples were 7.8% and 13.3% over the course of 0–50 F-T cycles, respectively. Similarly, for WP20 and NP20 samples, the corresponding loss rates were 12.5% and 19.4%, respectively. Furthermore, the compressive strength loss rates of the WP22 and NP22 specimens were 21.2% and 38.6%, respectively. In conclusion, 3.5 wt% NaCl solution will decrease the performance of CGPC F-T resistance and increase the loss rate of the compressive strength of CGPC. The compressive strength and compressive strength loss of CGPC are similar to that of pervious concrete with a natural basalt coarse aggregate. The loss rate of wp18 and wp20 after 100 F-T cycles is lower than that of natural basalt coarse aggregate pervious concrete [30].

### 3.4. The Permeability Coefficient

**Figure 11:**
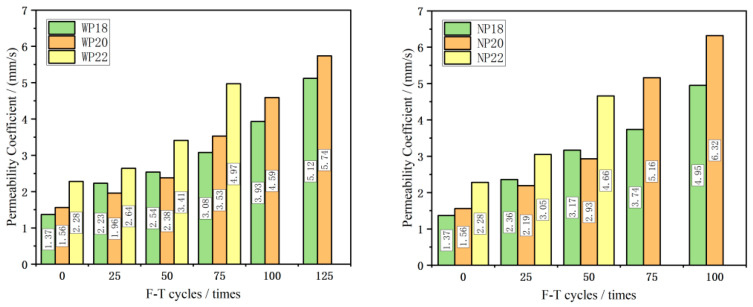
The permeability coefficient of CGPC after F-T cycles.

**Figure 12:**
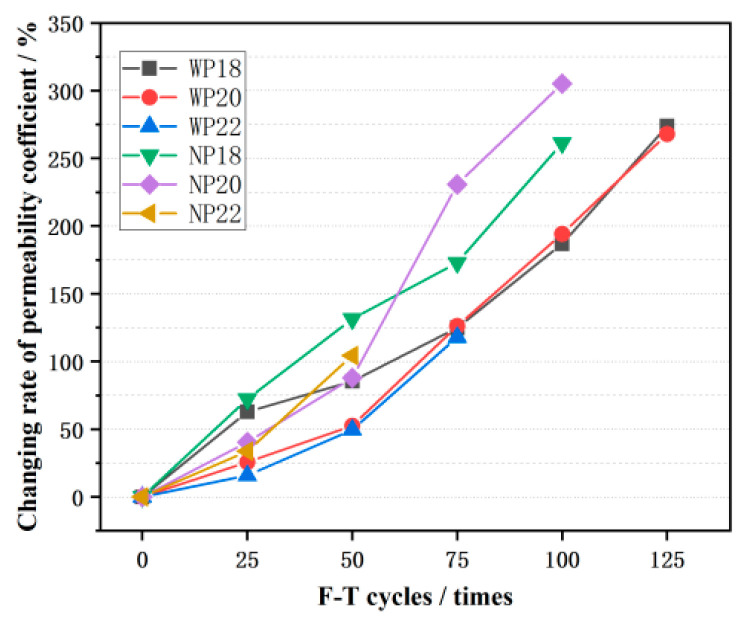
Change rate in the permeability coefficient of CGPC after F-T cycles.

The permeability coefficient of CGPC was tested using a self-made permeability coefficient tester, and the results are shown in Figure 11. As the number of F-T cycles increased, the permeability coefficient of CGPC also increased. Before the F-T process began, the permeability coefficients of CGPC with a designed porosity of 18%, 20%, and 22% were 1.37 mm/s, 1.56 mm/s, and 2.28 mm/s, respectively; thus, the permeability coefficient was positively correlated with the internal pores, which demonstrated that, as the F-T process continued, the internal structure of the specimen continued to deteriorate and the internal pores continued to expand. When CGPC was damaged by F-T, large areas of aggregates fell off in the cube specimens, and only the main body integrity could be maintained. At this time, the permeability coefficient reached its highest value. It can be noted from Figure 12 that the change in the permeability coefficient of the NP20 group’s specimens is the most conspicuous, with 100 F-T cycles. CGPC specimens in groups WP18, WP20, and WP22 were damaged, and the number of F-T cycles that obtained the highest values were 125 times, 125 times, and 75 times, respectively, which is more than the CGPC specimens in the NP18, NP20, and NP22 groups. This showed that the effect of salt water on the permeability coefficient of CGPC in the F-T environment was higher than that in water.

The rate of the F-T failure of concrete structures is connected to the rate of increase in the internal saturation of concrete. In F-T environments, 3.5 wt% NaCl solution is more prone to migration, and the increase in the internal saturation of saline concrete is faster than water. Its internal freezing pressure is also higher than that of concrete in water F-T environments, which has a more significant adverse impact on concrete specimens. On the other hand, some NaCl solutions entering the interior of coal gangue permeable concrete will react with the cement slurry, generating CaCl_2_. The solubility of CaCl_2_ is much higher than that of Ca(OH)_2_, which can also deteriorate the PC structure. Therefore, the rate of the F-T failure of CGPC in the salt solution is faster than that of CGPC in water.

### 3.5. The Relative Dynamic Elastic Modulus (RDEM)

**Figure 13:**
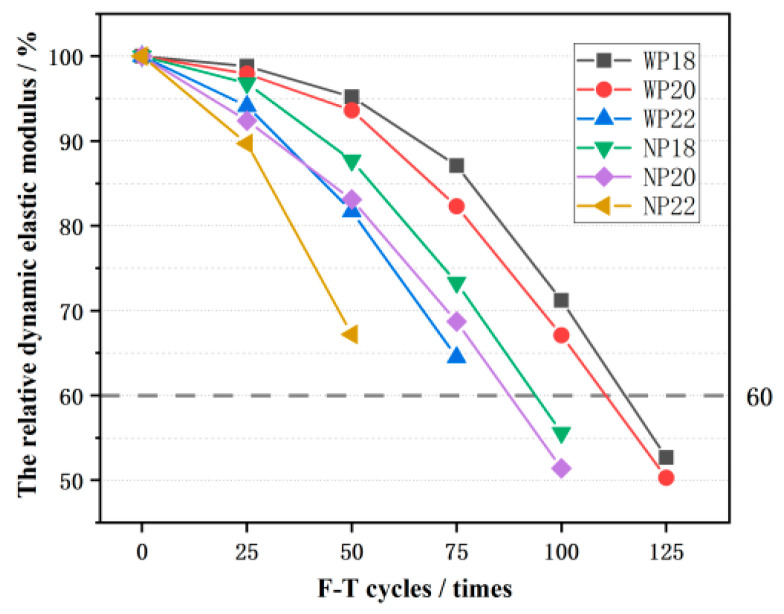
The relative dynamic elastic modulus of CGPC under F-T cycles.

RDEM is a significant indicator that reflects the PC damage’s state. When the weak surface of CGPC is broken and damaged, the part that still maintains the integrity of the main structure is tested, as shown in Figure 13. At the beginning of the F-T test, the RDEM of the CGPC declined relatively gently. The decline amplitude of the RDEM of CGPC increased by degree. As the number of F-T cycles increased, the deterioration degree of the CGPC samples became increasingly more severe during the continuous F-T process. The RDEM of the CGPC samples of NP18 and NP20 were lower than 60% after 100 F-T cycles, which reached the failure standard and caused F-T failure. The CGPC specimens of WP18 and WP20 reached the F-T failure standard during 100–125 F-T cycles. The F-T times of the CGPC samples subjected to 3.5 wt% NaCl solution were shorter than that in water, indicating that 3.5 wt% NaCl solution will aggravate the deterioration of CGPC specimens during the F-T process. Additionally, the RDEM of CGPC in the same F-T condition decreased with an increase in design porosity. After 75 F-T cycles, the RDEM of WP18, WP20, and WP22 were 87.1%, 82.3%, and 64.5%, respectively; however, that of NP18, NP20, and NP22 were 87.7%, 83.1%, and 67.2%, respectively. It is obvious that, when CGPC is placed in 3.5 wt% NaCl solution, the RDEM declines more quickly than in water during the F-T process, which means that 3.5 wt% NaCl solution will decrease the performance of CGPC F-T resistance and accelerate the deterioration of the CGPC specimen. After 100 freeze–thaw cycles, the RDEM of pervious concrete with a coarse aggregate of limestone (diameter 2.5–10 mm) in a water-frozen environment and salt-frozen environment decreased by 7.9% and 11.4%, respectively, both of which were much smaller than CGPC. However, the descending rule in the water-frozen environment and salt-frozen environment is similar to CGPC [4].

## 4. Model of Compressive Strength of CGPC after F-T Cycles

The GM (1,1) model can use differential equations to construct a data sequence without any regularity and make predictions. It is advantageous because it requires very little raw data, and that a high-precision prediction model can be constructed through at least four data points [41]. The calculation is simple and easy to verify. In response to the problem of the poor prediction accuracy of the GM (1,1) model when the degree of data dispersion is large, residual correction is introduced to establish a grey residual model. On the basis of using the Markov process to determine the sign of the residual correction value of the predicted value, the simulated and predicted values obtained using the grey residual model are corrected [42]. A grey residual–Markov model was established and utilized to forecast the compressive strength of CGPC after the 150th, 175th, and 200th F-T cycles.

### 4.1. Model Establishment Method

#### 4.1.1. GM (1,1) Model Establishment

The original data series representing the compressive behavior of CGPC with *X*^(0)^, ${X^{\left(0\right)}=\{X}^{\left(0\right)}(1),\;X^{\left(0\right)}(2),\;\ldots ,\;X^{\left(0\right)}(n)\}$, and the cumulative sequence $X^{\left(1\right)}$ is obtained by adding up the sequence $X^{\left(0\right)}$.

(7) $${X^{\left(1\right)}=\{x}^{\left(1\right)}(1),\;x^{\left(1\right)}(2),\ldots \;,\;x^{\left(1\right)}(n)\},$$

The sequence of adjacent means $Z^{\left(1\right)}$ is generated using the following formula [32]

(8) $$z^{\left(1\right)}(k)=\frac{1}{2}x^{\left(1\right)}(k)+x^{\left(1\right)}(k-1),\;k=2,\;3,\ldots ,\;n,$$

where $Z^{\left(1\right)}$ is ${\{z}^{\left(1\right)}(1),\;z^{\left(1\right)}(2),\ldots \;,\;z^{\left(1\right)}(n)\}$.

Establish an equation

(9) $${dX}^{\left(1\right)}/dt+aX^{\left(1\right)}=b,$$

where *a* and *b* can be calculated using the following formula

(10) $${\left[a,b\right]}^{T}={\left(B^{T}B\right)}^{-1}B^{T}Y,Y=\left[\begin{array}{c} \begin{array}{c} X^{\left(0\right)}(1)\\ X^{\left(0\right)}(2)\\ \vdots \end{array}\\ X^{\left(0\right)}(n) \end{array}\right],B=\left[\begin{array}{c} \begin{array}{cc} {-z}^{\left(1\right)}(2) & 1 \end{array}\\ \begin{array}{cc} {-z}^{\left(1\right)}(3) & 1 \end{array}\\ \vdots \\ \begin{array}{cc} {-z}^{\left(1\right)}(n) & 1 \end{array} \end{array}\right],$$

By solving the above differential model, the GM (1,1) model is obtained as follows:

(11) $${x^{\left(1\right)}(k+1)=(x}^{\left(0\right)}(1)-\frac{b}{a})e^{-ak}+\frac{b}{a},\;k=1,\;2,\;3,\ldots ,\;n,$$

(12) $${x^{\left(0\right)}(k+1)=x}^{\left(1\right)}(k+1)-x^{\left(1\right)}(k)={(1-e^{a})(x}^{\left(0\right)}(1)-\frac{b}{a})e^{-ak},\;k=1,\;2,\;3,\ldots ,\;n,$$

#### 4.1.2. Grey Residual Model

The residual sequence ${\varepsilon_{1}}^{\left(0\right)}$ is established using the following formula [35]

(13) $$\;{{\varepsilon_{1}}^{\left(0\right)}=\{\varepsilon_{1}}^{\left(0\right)}(2),\;{\varepsilon_{1}}^{\left(0\right)}(3),\ldots \;,\;{\varepsilon_{1}}^{\left(0\right)}(n)\},$$

(14) $${{\varepsilon_{1}}^{\left(0\right)}(k)=X}^{\left(0\right)}(k)-x^{\left(0\right)}(k),\;\;k=2,\;3,\ldots ,\;n,$$

Take the absolute value of ${\varepsilon_{1}}^{\left(0\right)}(k)$ and obtain $\varepsilon^{\left(0\right)}$,

(15) $${\varepsilon^{\left(0\right)}=\{\varepsilon }^{\left(0\right)}(2),\;\varepsilon^{\left(0\right)}(3),\ldots \;,\;\varepsilon^{\left(0\right)}(n)\},\;\;k=2,\;3,\ldots ,\;n,$$

GM (1,1) modeling was carried out for $\varepsilon^{\left(0\right)}$. The specific method is the same as in Section 4.1.1 above, and the following prediction model was obtained

(16) $${\varepsilon^{\left(1\right)}(k+1)=(\varepsilon }^{\left(0\right)}(2)-\frac{b_{e}}{a_{e}})e^{-a_{e}k}+\frac{b_{e}}{a_{e}},\;k=1,\;2,\;3,\ldots ,\;n\},$$

Through inverse accumulated generating operation of the following formula, the residual correction value $\varepsilon^{\left(0\right)}$ was obtained,

(17) $${\varepsilon^{\left(0\right)}(k+1)=\varepsilon }^{\left(1\right)}(k+1)-\varepsilon^{\left(1\right)}(k)={(1-e^{a})(\varepsilon }^{\left(0\right)}(2)-\frac{b_{e}}{a_{e}})e^{-a_{e}k},\;k=2,\ldots \ldots ,\;n,$$

$\varepsilon^{\left(0\right)}$ is used to correct $X^{\left(0\right)}$ by using the following formula:

(18) $${{x_{\varepsilon }}^{\left(0\right)}(k+1)=x}^{\left(0\right)}(k+1)\pm \varepsilon^{\left(0\right)}(k+1),\;k=2,\;3,\ldots ,\;n,$$

The above formula is called the grey residual GM (1,1) model, referred to as the grey residual model.

#### 4.1.3. Grey Residual–Markov Model

Based on the concept that “the system state at every moment only subjected to the state of the previous moment, and is irrelevant to its past history”, the Markov process is used to study the system state and the transition of the system state; it then predicts the prospective development tendency of the system state in accordance with the transition probability between the system states (that is, the possibility of switching from one state to another).

The specific steps of using the Markov process for determining the sign of residual correction at a certain time in the future are as follows:

Determine the status. Define two states, where state 1 means that the residual is plus and state 2 means that the residual is minus.

In accordance with the positive and negative states of the residual sequence, the state transition probability matrix *P* is calculated using the following formula [36],

(19) $$P=\left[\begin{array}{cc} p_{11} & p_{12}\\ p_{21} & p_{12} \end{array}\right],$$

where the element $p_{nm}$ means the probability of transferring from state *n* to state *m*, and $p_{nm}$ can be calculated using the following formula,

(20) $${\;p}_{nm}=\frac{{\;s}_{nm}}{s_{n}},\;n=1,2;\;m=1,\;2,$$

where the element $p_{nm}$ represents the times of transferring from state *n* to state *m*, and $s_{n}$ is the frequency of the appearance of state *n*.

Determine the current state vector. $s^{\left(0\right)}=({s_{1}}^{\left(0\right)},\;{s_{2}}^{\left(0\right)})$ is the initial state vector, ${s_{1}}^{(0)\;}$ is the probability of being in state 1, and ${s_{2}}^{\left(0\right)}$ is the probability of being in state 2.

According to the state transition formula shown below, the result of the state transition at the next time can be calculated [35].

(21) $$s^{\left(t\right)}=s^{\left(0\right)}\cdot P,$$

The state with high probability in $s^{\left(t\right)}$ is taken as the state of the residual correction value at *t* time t (i.e., the plus or minus sign). If the probabilities of the two states are equal, the state of the residual correction value at a previous time is taken as the state of the residual correction value at *t* time.

#### 4.1.4. The Relative Error

The formula of the relative error is as follows:

(22) $${∆x=\left|{\;X}^{\left(0\right)}(k)-x^{\left(0\right)}(k)\right|/x}^{\left(0\right)}(k),\;k=1,\;2,\;3,\ldots ,\;n,$$

### 4.2. Model Establishment of Compressive Strength of CGPC after F-T Cycles

**Figure 14:**
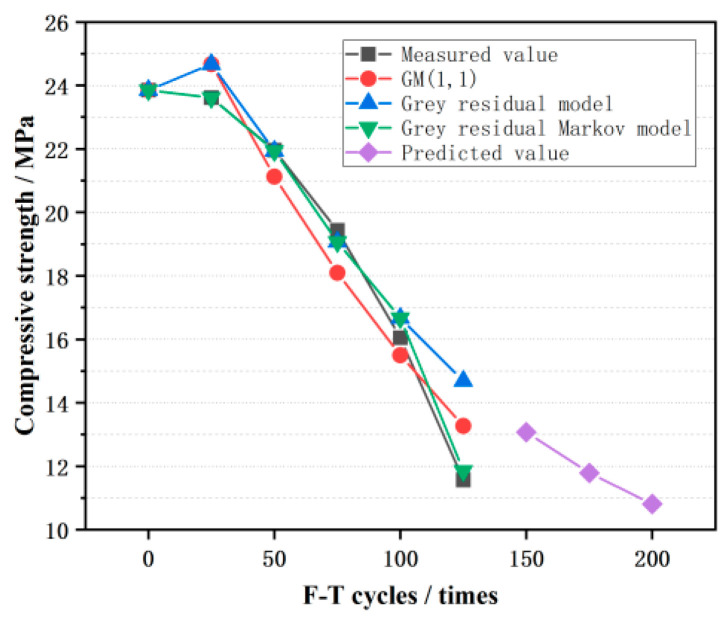
Comparison chart of measured values, simulated values, and predicted values of CGPC.

Considering the performance of CGPC in the F-T process, CGPC with a design porosity of 18% was chosen, and three different prediction models were established for its compressive strength in water during the F-T cycle process. The detailed calculation steps are as follows:

(1) Constructing the original data series:
  (23) $$X^{\left(0\right)}=(23.85,\;23.61,\;21.96,\;19.43,\;16.05,\;11.57),$$
(2) Obtaining the cumulative sequence $X^{\left(1\right)}$ and the sequence of adjacent means $Z^{\left(1\right)}$
  (24) $$X^{\left(1\right)}=(23.85,\;47.46,\;69.42,\;88.85,\;104.9,\;116.47),$$
  (25) $$Z^{\left(1\right)}=(35.655,\;58.44,\;79.135,\;96.875,\;110.685),$$
(3) Calculating parameters *a* and *b* via MATLAB R2023b
  (26) a=0.1550, b=30.3269,
(4) The GM (1,1) of the compressive strength CGPC is as follows:
  (27) $$x^{\left(0\right)}(k+1)=28.805e^{-0.155k},\;k=1,\;2,\;3,\ldots ,\;n,$$
(5) $\varepsilon^{\left(0\right)}$, $\varepsilon^{\left(1\right)}$, and ${x_{\varepsilon }}^{\left(0\right)}$ and the relative error of the grey residual model are listed in Table 6.
(6) The relative value of residuals is used to divide it into two states: $E_{1}$(−∞, 0) and $E_{2}$(0, ∞), and the transition probability matrix is calculated as per the state of the residual relative value sequence.
  (28) $$P=\left[\begin{array}{cc} 2 & 1\\ 1 & 0 \end{array}\right],$$
(7) The value of the grey residual–Markov model can be further calculated using the state of the residual relative value sequence, as shown in Table 7 and Figure 14, as well as the other model and its relative error.

### 4.3. Model Validation and Prediction

As shown in Table 6, the mean relative error of the grey residual–Markov model was minimal. In addition, in Figure 14, the curve of the grey residual–Markov model fits well with the measured results. Therefore, this model was applied to predict the compressive strength of CGPC after the 150th, 175th, and 200th F-T cycles, and the result is presented in Figure 14.

To sum up, the simulated results of the grey residual–Markov model fit well with the measured results. This model can simulate the compressive behavior of CGPC during F-T cycles and predict future compressive strength. However, there are only a few experimental data that can be utilized to verify this model in other literature [42,43,44]. Thus, the model is currently only valid for the measured results of this study. However, more tests will be performed to prove the accuracy of this model in the near future.

## 5. Conclusions

This study is the first to conduct experimental research on the behaviors of CGPC under F-T cycles in different F-T environments, exploring its variation patterns and establishing a prediction model using the grey residual–Markov theory. In this paper, the main conclusions were obtained as follows:

As the F-T cycle continues, the apparent deterioration of CGPC specimens gradually becomes severe. During the freeze–thaw cycle, the specimen undergoes the exposure of aggregates, the detachment of cement slurry and CGAs at edges and corners, the extensive detachment of CGAs and cement slurry, and, finally, the fracture and failure of the specimen. In 3.5 wt% NaCl solution, F-T damage occurs earlier than in water.

In F-T conditions involving both water and a 3.5 wt% NaCl solution, the weight of CGPC specimens consistently declined until they reached a point of deterioration and fracturing, resulting in a substantial loss in mass. When all other variables were held constant, the rate of mass loss for CGPC specimens in water was lower compared to those in the 3.5 wt% NaCl solution. Additionally, an increase in the designed porosity of the specimen corresponded to a higher rate of mass loss.

With an increase in the number of F-T cycles, the compressive strength and RDEM of CGPC maintained a downward trend, and they decreased more dramatically in 3.5 wt% NaCl solution than in water. The specimens with a larger designed porosity have a greater compressive strength loss rate and lower RDEM.

The permeability coefficient of the specimen increases as the F-T cycles continue. In the equal F-T process, the higher the design porosity of CGPC, the higher the permeability coefficient of the specimen. When other F-T conditions are the same, the permeability coefficient of the CGPC specimens is higher in 3.5 wt% NaCl solution than in water.

The GM (1,1) model, grey residual model, and grey residual–Markov model were established based on the grey theory and Markov process. By comparing the mean relative error of the three models, the mean relative error of the grey residual–Markov model was 2.08%, which was minimal, and this model had good accuracy. Therefore, it was used to predict the compressive behavior of CGPC during the F-T process and has particular potential for engineering practices. However, this method also has limitations, and the error of a few correction results will be larger when the Markov process is used for symbol correction. This is because the Markov process determines the positive and negative of the subsequent residual according to the probability of the positive and negative residual, which has a certain regularity. However, the changes in the data in some experimental groups may be irregular and random; therefore, the improvement of the Markov model still needs to be further discussed.

## Figures and Tables

**Table 1:** Chemical analysis of cement.

Chemical Composition	CaO	SiO_2_	Al_2_O_3_	Fe_2_O_3_	MgO	SO_3_	Cl^−^	Others
(%)	56.77	20.86	5.9	3.61	3.5	2.43	0.02	6.91

**Table 2:** Properties of cement.

Specific Surface Area (m^2^/kg)	Density (g/cm^3^)	Compressive Strength (MPa)		Flexural Strength (MPa)		Initial Setting Time (min)	Final Setting Time (min)	Stability
		3d	28d	3d	28d			
382	3.1	32.2	61.3	6	8.6	149	211	Qualified

**Table 3:** Basic physical properties of coal gangue aggregate.

Particle Size (mm)	Loose Bulk Density (kg/m^3^)	Compacted Bulk Density (kg/m^3^)	Apparent Density (kg/m^3^)	Void Content (%)	Moisture Content (%)	Water Absorption (%)
9.5~16	1206	1405	2818	44.7	0.67	2.68

**Table 4:** Chemical analysis of coal gangue aggregate.

Chemical Composition	SiO_2_	Al_2_O_3_	CO_3_	Fe_2_O_3_	K_2_O	CaO	Na_2_O	MgO	Others
(%)	61.7	19.11	5.83	4.16	3.04	2.35	2.28	0.64	0.89

**Table 5:** Mix proportion of concrete.

Sample Designation	Designed Porosity	Coal Gangue Aggregate (kg/m^3^)	Cement (kg/m^3^)	Water (kg/m^3^)	PC Reinforcement Agent (kg/m^3^)
P18	18%	1376.90	540.98	156.88	17.31
P20	20%	1376.90	508.33	147.41	16.27
P22	22%	1376.90	475.68	137.95	15.22

**Table 6:** Grey residual model of compressive strength of CGPC under F-T cycles.

F-T Cycles	Measured Value (MPa)	${\varepsilon_{1}}^{\left(0\right)}$	$\varepsilon^{\left(0\right)}$	$\varepsilon^{\left(1\right)}$	${x_{\varepsilon }}^{\left(0\right)}$
0	23.85	/	/	/	23.85
25	23.61	−1.06	1.06	1.06	24.67
50	21.96	−0.83	0.83	0.81	21.94
75	19.43	−1.33	1.34	0.98	19.07
100	16.05	−0.53	0.55	1.17	16.67
125	11.57	−1.66	1.70	1.42	14.69

**Table 7:** Simulated values and their relative error of CGPC.

Measured Value (MPa)	Value of GM (1,1) Model		Value of Grey Residual Model		Value of Grey Residual Markov Model	
	Compressive Strength (MPa)	Relative Error	Compressive Strength (MPa)	Relative Error	Compressive Strength (MPa)	Relative Error
23.85	23.85	0.00%	23.85	0.00%	23.85	0.00%
23.61	24.67	4.49%	24.67	4.49%	23.61	0.00%
21.96	21.13	3.79%	21.94	3.69%	21.94	0.10%
19.43	18.09	6.87%	19.07	5.02%	19.07	1.85%
16.05	15.50	3.45%	16.67	7.32%	16.67	3.87%
11.57	13.27	14.71%	14.69	12.23%	11.86	2.48%
Mean relative error	6.66%		6.55%		2.08%	

## Data Availability

Data are contained within the article.
